# P02.100. Adjunctive naturopathic care in type 2 diabetes: patient-reported and clinical outcomes

**DOI:** 10.1186/1472-6882-12-S1-P156

**Published:** 2012-06-12

**Authors:** R Bradley, K Sherman, S Catz, E Oberg, C Calabrese, D Cherkin

**Affiliations:** 1 Bastyr University, Kenmore, USA; 2 Group Health Research Institute, Seattle, USA; 3 Naturopathic Physicians Research Institute, Portland, USA

## Purpose

We conducted a one-year prospective cohort study to determine the promise and feasibility of a randomized clinical trial of adjunctive naturopathic care (ANC) for type 2 diabetes (T2D).

## Methods

40 patients from Group Health Cooperative (GHC), a large integrated healthcare system in Washington State, with existing T2D and hemoglobin A1c values between 7.5-9.5%, were recruited to receive up to eight free visits of ANC over one year in addition to their usual care. Changes from baseline in self-care, self-efficacy and mood based on validated instrument scores were compared within the intervention group after 6- and 12- months. Clinical risk factors were also compared within group, and to a control group generated using electronic medical records (n=329), at the end of one-year.

## Results

Significant improvements were measured in composite scores of the Summary of Diabetes Self-Care Activities (SDSCA) instrument for diet (+20% mean score, p=0.001), exercise (+28% mean score, p=0.02) and glucose testing (+45% mean score, p=0.001). Significant changes were also measured in depression scores measured by PHQ-8 (-33% mean score, p=0.001) and improved self-efficacy measured by the Self-Efficacy Scale (+25% mean score, p=0.0001). Many of the observed changes in self-care corresponded to the period of more intensive ANC utilization, i.e. the first six months, however improvements in glucose testing, self-efficacy and improved mood scores persisted at 1-year (+24% mean score, p=0.002; +43% mean score, p=0.003; and -33% mean score, p=0.005 respectively). Changes in clinical risk factors were small during the period of ANC, though hemoglobin A1c (HbA1c) improved significantly within group (-0.90%, p=0.02) and compared to electronic controls (-0.51%, p=0.07) at 6-months.

## Conclusion

Patients with inadequately controlled T2D improved in their self-care behaviors, self-efficacy, mood and clinical risk during the period of ANC. Clinical trials are feasible and warranted.

